# School-Aged Children With Higher Reflective Functioning Exhibit Lower Cardiovascular Reactivity

**DOI:** 10.3389/fmed.2018.00196

**Published:** 2018-07-06

**Authors:** Jessica L. Borelli, Karin Ensink, Kajung Hong, Alexandra T. Sereno, Robert Drury, Peter Fonagy

**Affiliations:** ^1^THRIVE Laboratory, Psychology and Social Behavior, University of California, Irvine, Irvine, CA, United States; ^2^Department of Psychology, University Laval, Quebec, QC, Canada; ^3^Wisconsin Institute for Discovery, Bainbridge Island, WA, United States; ^4^University of Wisconsin-Madison, Madison, WI, United States; ^5^ReThink Health, Cambridge, MA, United States; ^6^Anna Freud National Centre for Children and Families & University College London, London, United Kingdom

**Keywords:** reflective functioning, mentalization, children, respiratory sinus arrhythmia, attachment

## Abstract

Despite extensive theorizing regarding the regulatory role of reflective functioning (RF), few studies have explored the links between RF and physiological indices of emotion regulation, and none have examined these associations in children. Further, while scholars contend that RF promotes resilience via enhanced ability to process emotional experiences, including those occurring in attachment relationships, this argument has seldom been tested empirically in children. In the current study, we explore the association between RF and physiological measures of emotion reactivity and regulation, as well as the interaction of RF and attachment insecurity. We test these associations by examining children's (*N* = 76; 8–12 years old) cardiovascular responses [respiratory sinus arrhythmia (RSA)] to a standardized paradigm designed to evoke reactions regarding the experience and expression of attachment-related needs. Children also completed a semi-structured attachment interview, which was later coded for children's attachment insecurity (operationalized as attachment dismissal and preoccupation) and RF. Our findings were largely consistent with theory and our hypotheses, suggesting that higher RF is associated with lesser cardiovascular reactivity (higher levels of RSA) during the stressor task and better recovery following the task. These links were especially strong for children with greater attachment preoccupation but did not vary as a function of children's levels of attachment dismissal. These findings contribute to developmental theory in suggesting that RF is closely linked to physiological emotion regulation in children.

## Introduction

Emotion serves an important role in orienting us to attend to internal or external stimuli (1, 2). Regulation of emotions, which involves conscious and unconscious processes (3, 4), is a key developmental milestone and transdiagnostic protective factor against psychopathology (5–7). Emotion is a multifaceted construct comprised of experiential, behavioral, and physiological components, with each factor revealing unique information (3). Measuring these different components has the potential to give insight into those unique streams of information. Physiological measures of emotion can be particularly useful in measuring autonomic arousal, a metric that is less susceptible to reporting biases or social desirability effects than other assessments (e.g., self-report). Heart rate variability (HRV), an index of the change in time invervals between heartbeats, is a measure of physiological reactivity that reflects the interplay of different physiological systems that enable us to adapt to challenges in the internal and external environment (8). Respiratory sinus arrhythmia (RSA) is a short-term measure of HRV that reflects the vagus nerve's influence on the slowing and speeding of the heart. RSA captures parasympathetic nervous system activation in response to environmental stimuli (9). Higher levels of task-related changes in RSA indicate lower levels of sympathetic reactivity (10–12). While the initial response to a stimulus indexes emotion reactivity, observed recovery or return to baseline RSA can be used to infer emotion regulation, as has been done in prior studies (13, 14).

## Attachment

Attachment security, or the felt sense that others will be responsive to one's expression of needs for comfort and support, is thought to develop as a result of a history of receiving sensitive care from attachment figures (15). The internal working model, a cognitive-affective schema that emerges from a history of interactions between infant and caregiver, contains important beliefs regarding the experience and expression of emotion (15): when children's expressions of emotional need have been met consistently with empathy and assistance in regulating emotion, children internalize the message that painful emotional experiences can be experienced, expressed, and resolved, resulting in optimal self-regulation of emotion later in development (16). In contrast, when children's needs have been rejected or ignored or when caregivers have responded inconsistently or with alarm to children's needs, children resort to defensive emotion regulation strategies, such as deactivation or hyperactivation, which while adaptive in the short-term, can result in negative outcomes over the long-term (16).

Decades of research substantiate this theorizing by documenting links between attachment security and emotion regulation in adults [e.g., (17, 18)]. Although middle childhood remains an understudied developmental phase with respect to attachment and its links with emotion (19), emerging evidence suggests that school-aged children with secure attachment have better emotion regulation than their insecure counterparts [e.g., (20, 21)].

The association between attachment and emotion regulation is thought to depend on early parent-child interactions involving physical/embodied regulation by the parent (22, 23). These interactions serve to calibrate the infant's developing stress regulation system so that over time, physiological *self*-regulation is established (24), with the presence of the parent needed and sought only in contexts of threat or higher levels of distress. In addition, expectancies regarding the parent's availability to respond to distress are reflected at a representational level. By middle childhood, attachment processes (1) have facilitated emotional regulation through their early physiological impact on the development of the stress regulation system; (2) continue to facilitate emotional regulation through the actual availability of, support from, and protection offered by attachment figures in times of distress (25); and (3) promote regulation at a representational level regarding the imagined responsiveness and trustworthiness of attachment figures and others in times of need (26). In line with this perspective, longitudinal evidence suggests that the quality of early parenting, through its impact on epigenetic regulation and DNA methylation, has long term implications on self-regulation and interpersonal processes into adulthood (27–29).

## Mentalization

As conceptualized by Fonagy et al. (24), *mentalization* refers to the process of interpreting the reactions of others in terms of psychological experience, imagining the mental states and intentions that underlie behavior, and being cognizant of one's own emotional reactions and their impact on others. Mentalization has been operationalized for research purposes as reflective functioning (RF). RF includes a self-focused dimension of one's own mental states, as well as an other-focused dimension concerning others' experiences (30–32).

Mentalization develops alongside attachment when infants are treated as individuals with minds and are responded to as if their behavior communicates something about their psychological experience (33). Through being treated as intentional agents, children discover their minds and come to think of themselves as having thoughts, desires, and feelings (34). Consistent with this argument, parental mentalization predicts school-aged children's own mentalization (30).

People with higher RF are more likely to have secure attachment (35), perhaps as a result of the association between sensitive parenting and parental mentalization, but the two constructs are not synonymous. Evidence suggests that adults with secure attachment are more likely to have higher RF (36–38), but the effect sizes obtained in these studies are not large, suggesting the distinctiveness of the two constructs. However, to date only one study has explored the links between attachment security and RF in children, finding that lower RF is associated with dismissing and disorganized, but not preoccupied attachment (Bizzi et al. in press).

## Mentalization and emotion regulation

Scholars contend that there is a bidirectional association between RF and emotion reactivity and regulation (24). Having access to representations, metacognitive reflection, and semantic processing regarding one's own emotions facilitates the ability to make sense of and modify emotional expeirence. Fonagy et al. (24) describe a particular type of early non-verbal communication involving marked mirroring of the infant's peak affects and ostensive cueing, suggesting that this type of interaction provides the infant with an external representation of his/her emotions that is important for the consolidation of an early sense of self (39). Thus, mentalization is thought to serve a powerful regulatory function, helping individuals make sense of their own and others' behaviors, thoughts, and feelings, and in so doing, create an environment in which emotions are viewed as predictable, meaningful, and controllable (24). At the same time, mentalization is impacted by emotional arousal—higher levels of arousal may inhibit effortful and deliberate RF (31, 40).

Despite the rich theorizing regarding the regulatory role of mentalization, the links between RF and emotion have seldom been examined empirically, and have not yet been explored in children. Although there is no direct evidence of the link between children's RF and children's emotion regulation, several studies provide indirect support for this association. First, there is evidence that mentalization in parents predicts parenting behavior that is associated with better emotion regulation in children. For instance, parents with higher RF engage in more sensitive (41–45) and less intrusive or frightening parenting than parents with lower RF (41, 46). We see this as consistent with the theorized association, given that more sensitive or controlled parenting may in and of itself be evidence of better emotion regulation (47). Second, in a series of studies on parental mentalization and school-aged children's emotional adjustment (48, 49), Gottman and colleagues find that parents who show greater awareness of their own and their children's emotions have children who exhibit better emotion regulation. Conversely, lower RF confers risk for a variety of forms of psychopathology, including autism, depression, psychosis, PTSD, eating disorders, substance abuse [for a review see (50–55)], as well as forms of psychopathology chiefly characterized by emotion dysregulation [e.g., borderline personality disorder; (56, 57)], suggesting that the two may be linked. Similarly, in adolescents, lower RF is a general risk factor for psychopathology, including borderline and narcissistic personality traits, as well as internalizing and externalizing symptoms (58). Finally, in children, lower RF is associated with more depressive, externalizing, and somatic symptoms [Bizzi et al., under revision, (59, 60)].

## RF as a resilience factor in the context of attachment

According to theory, mentalization can assist individuals in the processing of life experiences, including those that occur in attachment relationships (24, 61). Mentalization can help individuals understand and make sense of their past experiences, which is thought to be central to resilience. This conceptualization of mentalization converges with the notion of resilience as a “reintegration of self that includes a conscious effort to move forward in an insightful integrated positive manner as a result of lessons learned from an adverse experience” [(62), p. 3].

In support of this argument, research finds that among adolescents who report having experienced parental neglect (adverse early experience), those with higher RF were less likely to be classified as having insecure attachment (outcome) compared to their lower-RF counterparts (63). Similarly, another study found that among parents with childhood experiences of maltreatment (adverse early experience), those with higher levels of RF regarding trauma were less likely to have infants who were disorganized in their attachment [outcome variable; (64)]. In the current study, we examine whether the link between RF and RSA is stronger among children with greater attachment insecurity (more attachment preoccupation or dismissal), who are likely to have experienced greater distress in the context of attachment related needs than children with lesser attachment insecurity, thereby assessing whether RF can promote resilience in the context of attachment experiences.

## Current investigation

We pursue two central aims—first, we test the concurrent associations between school-aged children's RF and their physiological reactivity to and recovery following a stressor task related to attachment needs. Second, we explore whether RF interacts with attachment insecurity in its associations with physiological reactivity and recovery.

To these ends, a community sample of school-aged children completed an attachment interview, which was later coded by independent teams of raters naïve to study hypotheses for attachment security and RF. Approximately 1 week later, children completed a standardized laboratory paradigm used in previous studies of attachment (65), in which they read hypothetical vignettes of other children encountering situations that are likely to evoke attachment-related needs (e.g., being sick, feeling afraid). During and following the presentation of these vignettes, we monitored children's RSA, which we used as measures of reactivity and regulation, respectively.

We tested the following hypotheses. First, consistent with prior work, we sought to replicate the association between higher attachment insecurity (dismissal and preoccupation) and lower RF. Second, we predicted that higher RF would be associated with lower reactivity and regulation (higher RSA during and following the stressor task). We followed this prediction with an exploratory test of the pathway between RF and emotion regulation, testing whether RSA during the stressor (emotion reactivity) mediates the association between RF and RSA following the stressor (emotion regulation). Third, we tested the theory that RF buffers the effects of attachment insecurity; we predicted that RF and attachment insecurity would interact in their association with RSA during (emotion reactivity) and following the stressor (emotion regulation), such that for children higher in attachment insecurity, higher RF would be more strongly associated with attenuated reactivity and regulation. Similar to above, we followed this hypothesis-driven prediction with an exploration of a moderated mediation model in which RF moderates the link between attachment insecurity and RSA following the stressor, as mediated by RSA during the stressor.

## Methods

### Participants

The protocol for this study was approved by the Institutional Review Board at Pomona College. Children (*N* = 76; 50% boys, *M*_*age*_ = 9.82, *SD*_*age*_ = 1.47) between the ages of 8 and 12 participated in this study of children's development. The principal investigator calculated the targeted sample size based on a power analysis using effect sizes obtained from her prior work on attachment in school-aged children, which suggested a sample size of *N* = 70 would be sufficient to detect an effect. The participants were recruited from the community through advertisements posted online, flyers, and word of mouth. The sample was racially/ethnically (40% of caregivers identified as Hispanic, 36% Caucasian, 13% African American, 4% Other, 1% Asian, and 1% Native American) and socioeconomically diverse (50% of families reported an annual income < $40,000; 8% >$120,000).

### Procedure

The study took place over two sessions occurring ~2 weeks apart. Caregivers provided consent to participate in a study, while children provided informed assent. Participants were informed that they could choose to opt out of any portion of the study at any time. Then children completed the Child Attachment Interview [CAI; (66)], from which two non-overlapping teams of coders scored children's attachment security and RF. On the second visit, children completed a laboratory stressor and recovery task during which we monitored their cardiovascular reactivity.

### Measures

#### Attachment security

Children completed the CAI, a semi-structured interview for 8–13 year olds, designed to assess the quality of their attachment to their caregivers. The interview consists of 19 questions about children's current and past experiences with their caregivers. Responses are coded on 8 scales (e.g., Idealization, Preoccupying Anger, Balance of positive/negative references to attachment figures), as well as on the Overall Narrative Coherence scale, a dimensional measure of attachment security (67). A certified CAI coder coded all interviews, with reliability performed on 16 randomly-selected cases coded by a second certified coder. The Intraclass Correlation Coefficients (ICC) for CAI scales ranged from 0.72 to 0.97. The average ICC across all CAI subscales was 0.87.

The CAI manual provides guidelines for using scale scores to place children into one of four best-fitting attachment classifications with respect to each caregiver: secure, dismissing, preoccupied, and disorganized. However, in line with the argument that attachment is best reflected using dimensional, rather than categorical, metrics (68, 69), we used factor analytically-derived scales of dismissing and preoccupied attachment. This procedure has previously been used with studies using the CAI (70–72). The details of the factor analysis conducted within the larger sample are reported elsewhere (73), but in brief, the analysis revealed the presence of two factors. Scales loading on the first factor (eigenvalue = 4.83) signified attachment dismissal, with high scores indicating high dismissal of attachment needs and idealization of relationships with caregivers. Scales loading on the second factor (eigenvalue = 1.86) signified preoccupation, with high scores indicating high involving/preoccupying anger (communalities above 0.70). A high level of inter-rater reliability was achieved on these factor scales, dismissal ICC = 0.92, preoccupation ICC = 0.96.

#### Reflective functioning

Children's RF was coded from their responses to the CAI using the Child Reflective Functioning Scale [CRFS; (30, 74)]. The CRFS is a modification of the Adult Reflective Functioning Scale [ARFS; (75)], which is used to measure RF on the Adult Attachment Interview [AAI; (76)]. The CRFS involves coding children's ability to articulate their own and others' internal experiences while describing current and past experiences with their caregivers. Coders rate RF on each CAI question; these scores are then averaged to create a global RF score. Although relatively recently developed, the CRFS has already shown promising psychometrics—the item-total correlations ranged from 0.57 to 0.79, and Cronbach's alpha was 0.94 (36). Due to our interest in children's general reflective capacities, in the current study we use children's global RF scores in analyses. In this study, internal consistency in RF scores across items was high, α = 0.96. The coder of all of the CAIs in this sample was trained by and was demonstrated to have excellent reliability with the developer of the CRFS measure (ICC = 0.92). The CRFS coder was unaware of all information regarding the children in the study (including their attachment classifications) and was not part of the attachment coding team.

The CRFS manual contains descriptions and examples of different levels and types of children's RF. Children's narratives are coded on an 11-point scale (1–9) descriptively anchored at six points in terms of their propensity to consider interpersonal interactions and personal reactions in mental state terms. To obtain a general indicator of children's RF (CRF-G), the mean RF of all the coded responses was used. The scale alpha was 0.94, and item-total correlations ranged from 0.57 to 0.79, confirming that the total score (CRF-G) could be used as a good indicator of overall RF. Because of theoretical considerations and previous findings with adults indicating that self- and other understanding may have distinct implications, self and other items were treated as separate scales. A factor analysis is not reported given that the sample was composed in part of children with histories of sexual abuse involving their fathers and that this may have had an effect on their mentalization regarding fathers that may be particular to this sample and would be unlikely to be replicated in other samples.

#### Laboratory stressor: distress vignettes paradigm

Children completed a standardized laboratory stressor task in which they were presented with multiple vignettes in text form regarding same-sex hypothetical children experiencing mildly emotionally and physically distressing situations (sadness, fear, sick, and hurt) on a computer screen (65). They were asked to reflect on their thoughts and feelings in reaction to each vignette (e.g., *[Child's name] hurt her/his knee when (s)he was playing basketball. It hurt all day long*). We used two different counterbalanced conditions to control for order effects of the presentation of the different situations (Order 1: hurt, sad, afraid, sick, neutral; Order 2: neutral, sick, hurt, sad, afraid). For each distressing situation, three vignettes were presented (order randomized within counterbalanced block) to represent increasing levels of severity of distress. All stimuli were presented using E-Prime. Prior data using this paradigm suggest that children experience significant increases in self-reported negative emotion in response to these vignettes (65).

#### Cardiovascular physiology

RSA data were collected before, while, and after the laboratory stressor was presented. Baseline RSA was collected while children sat quietly and watched a 290 s nature video. During the laboratory task, RSA-stressor was collected while each of the vignette (“story”) and reflection periods were presented (60 s for each vignette). Following each distress block (e.g., afraid), which included the presentation of three separate vignettes and reflection periods, children completed a 30 s RSA recovery period during which they were asked to sit quietly and wait until the next “story” appeared on the screen. Thus, for the purposes of this study, we considered RSA recordings taken during the vignettes to be measures of reactivity, whereas we considered RSA measures taken during the recovery periods following each block of vignettes to be measures of regulation.

We collected HRV data using disposable Mindware 1.5-in foam EKG electrodes with 7% chloride wet gel and touchproof snap leads, which were connected to a BioNex 8 slot chassis equipped with an impedance cardiograph (Mindware Technologies, Gahanna, OH). Data were collected using BioLab 2.5 acquisition software and were later edited for peak errors and noise using BioLab HRV 2.0 application (Mindware Technologies, Gahanna, OH). Prior to conducting data analysis, we computed mean RSA across the baseline assessment, and the reactivity and recovery sessions of the four distressing vignette types.

### Data analytic plan

To evaluate our hypotheses, we used hierarchical linear regressions in which we controlled for children's age and gender on an initial step. For analyses involving moderation, mediation, and moderated mediation, we used Hayes' PROCESS macro (77). In analyses in which reactivity and recovery levels of RSA were the dependent variables, we included baseline RSA as an additional covariate.

## Results

### Descriptive statistics

Descriptive statistics for key variables, overall and by child gender, are reported in Table 1. Independent samples *t-*tests revealed that girls were significantly older, less dismissing, and higher in RF than boys. Zero-order correlations indicated that older children had higher RF (*r* = 0.40, *p* < 0.001) and higher RSA-recovery (*r* = −0.25, *p* = 0.03; see Table 2). Children with more dismissing attachment had lower RF (*r* = −0.58, *p* < 0.001).

**Table 1:** Descriptive statistics of key variables by children's gender.

**Measures**	**Total (*N =* 76)**	**Boys (*n =* 38)**	**Girls (*n =* 38)**	**Gender differences *t***
	***M* (*SD*)**	***M* (*SD*)**	***M* (*SD*)**	
Age	9.82 (1.47)	9.24 (1.38)	10.39 (1.33)	−3.72^***^
Attachment dismissal^a^	0.05 (1.00)	0.36 (1.01)	−0.27 (0.88)	2.89^**^
Attachment preoccupation^b^	0.11 (0.98)	0.10 (0.97)	0.12 (1.00)	−0.11
RF	3.09 (0.84)	2.71 (0.69)	3.47 (0.80)	−4.45^***^
RSA-baseline	6.85 (1.24)	6.63 (1.38)	7.08 (1.05)	−1.60
RSA–stressor	6.75 (0.90)	6.68 (0.94)	6.83 (0.85)	−0.70
RSA–recovery	6.79 (0.90)	6.77 (0.91)	6.80 (0.90)	−0.16

RF, Reflective functioning;

** p < 0.01.

*** p < 0.001.

a *Attachment dismissal, Factor analytically derived dismissing attachment score (Child Attachment Interview); Higher score means highly dismissing*.

b *Attachment preoccupation, Factor analytically derived preoccupied attachment score (Child Attachment Interview); Higher score means highly preoccupied*.

**Table 2:** Correlation matrix for key variables.

**Variable**	**1**	**2**	**3**	**4**	**5**	**6**	**7**	**8**
1. Age	–							
2. Gender	0.40^***^	–						
3. Attachment dismissal^a^	−0.18	−0.32^**^	–					
4. Attachment preoccupation^b^	0.03	0.01	−0.04	–				
5. RF	0.40^***^	0.46^***^	−0.58^***^	−0.10	–			
6. RSA-baseline	−0.01	0.18	0.02	−0.07	−0.05	–		
7. RSA–stressor	−0.22	0.08	−0.09	−0.02	0.10	0.58^***^	–	
8. RSA–recovery	−0.25^*^	0.02	−0.04	−0.06	0.13	0.53^***^	0.84^***^	–

RF, Reflective functioning; Gender coding: 1, boys; 2, girls;

* p < 0.05,

** p < 0.01,

*** p < 0.001.

a *Attachment dismissal, Factor analytically derived dismissing attachment score (Child Attachment Interview); Higher score means highly dismissing*.

b *Attachment preoccupation, Factor analytically derived preoccupied attachment score (Child Attachment Interview); Higher score means highly preoccupied*.

Based on the results of these preliminary analyses, we controlled for children's age and gender in all subsequent analyses.

### Hypothesis 1. association between children's RF and attachment security

After controlling for children's age and gender (*R*^2^ = 0.27, *p* < 0.001), attachment dismissal was negatively associated with children's RF (Δ*R*^2^ = 0.22, *b* = −0.40, *SE* = 0.08, *p* < 0.001; see Table 3). In a subsequent analysis, when we controlled for attachment preoccupation (*R*^2^ = 0.28, *p* < 0.001), attachment dismissal was still negatively associated with children's RF (Δ*R*^2^ = 0.21, *b* = −0.40, *SE* = 0.08, *p* < 0.001), but preoccupation was not.

**Table 3:** Child attachment dismissal associated with child RF.

	**Dependent variable: child RF**			
**Step**	***b***	**SE**	**β**	**CI**
Step 1 R^2^	0.27^***^			
Constant	0.79	0.57		[−0.34, 1.92]
Age	0.14^*^	0.06	0.25	[0.02, 0.27]
Gender	0.60^**^	0.18	0.36	[0.24, 0.96]
Step 2 ΔR^2^	0.22^***^			
Attachment dismissal^a^	−0.40^***^	0.08	−0.48	[−0.55, −0.25]
Attachment preoccupation^b^	−0.11	0.07	−0.13	[−0.25, 0.04]

RF, Reflective functioning;

* p < 0.05;

** p < 0.01

*** *p < 0.001*.

a *Attachment dismissal = Factor analytically derived dismissing attachment score (Child Attachment Interview); Higher score means highly dismissing*.

b *Attachment preoccupation = Factor analytically derived preoccupied attachment score (Child Attachment Interview); Higher score means highly preoccupied*.

### Hypothesis 2. association between children's RF and RSA

Table 4 depicts the results of two hierarchical linear regressions testing the association between children's RF and RSA-stressor, as well as children's RF and RSA-recovery. After controlling for children's age, gender, and baseline RSA (*R*^2^ = 0.38, *p* < 0.001), children's RF was significantly positively associated with RSA-stressor (Δ*R*^2^ = 0.05, *b* = 0.28, *SE* = 0.11, *p* = 0.01; Hypothesis 2a). Thus, in support of Hypothesis 2, higher RF was associated with lesser reactivity.

**Table 4:** Hierarchical regressions examining associations between children's RF, RSA-stressor and RSA-recovery.

	**Dependent variable: RSA–stressor**				**Dependent variable: RSA–recovery**			
**Step**	***b***	***SE***	**β**	**CI**	***b***	***SE***	**β**	**CI**
Step 1 R^2^	0.38^***^				0.34^***^			
Constant	5.22^***^	0.73		[3.76, 6.67]	5.62^***^	0.76		[4.10, 7.13]
Age	−0.15^*^	0.06	−0.24	[−0.27, −0.02]	−0.15^*^	0.06	−0.25	[−0.28, −0.02]
Gender	0.13	0.18	0.07	[−0.23, 0.50]	0.04	0.19	0.02	[−0.34, 0.42]
RSA–Baseline	0.41^***^	0.07	0.56	[0.27, 0.54]	0.38^***^	0.07	0.52	[0.24, 0.52]
Step 2 ΔR^2^	0.05^*^				0.09^**^			
RF	0.28^*^	0.11	0.26	[0.06, 0.51]	0.37^**^	0.11	0.34	[0.14, 0.60]

*RF, Reflective functioning*.

* *p < 0.05*.

** *p < 0.01*.

*** *p < 0.001*.

Second, after controlling for the same set of covariates (*R*^2^ = 0.34, *p* < 0.001), children's RF was significantly positively associated with children's RSA-recovery *(*Δ*R*^2^ = 0.09, *b* = 0.37, *SE* = 0.11, *p* = 0.002; Hypothesis 2b). Therefore, in support of our hypothesis, higher RF was associated with greater parasympathetic activation during the recovery period.

#### Exploratory mediation

**Figure 1:**
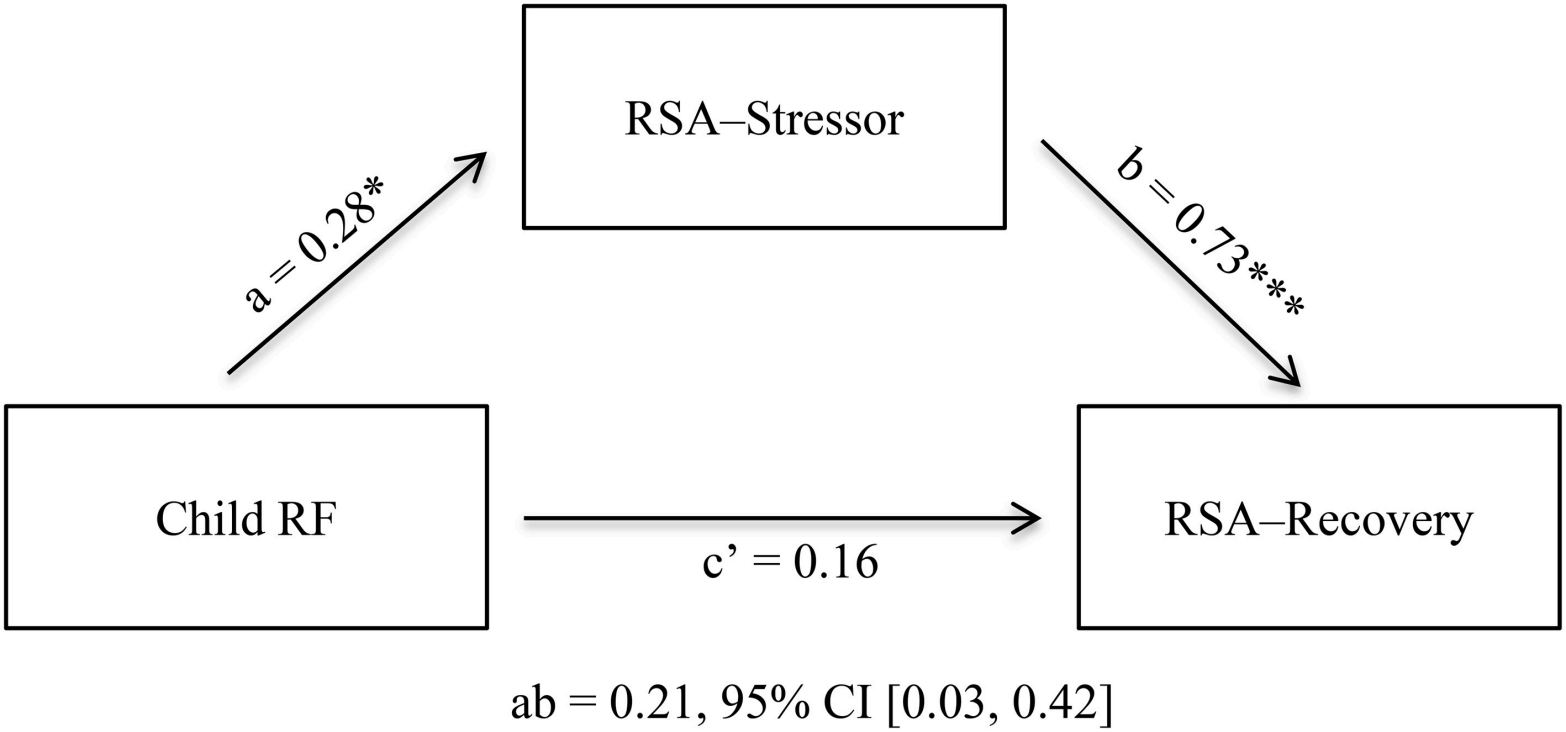
RSA-stressor as a mediator for the association between Child RF and RSA-recovery. Figure shows unstandardized *b* values. Analysis includes the following covariates (not pictured here): child age, child gender, RSA-Baseline. RF, Reflective functioning; ^*^p < 0.05. ^***^p < 0.001.

Figure 1 presents the results of a hierarchical regression testing the mediation model. PROCESS Model 4 revealed that after controlling for children's age, gender, and baseline RSA in the first step (*R*^2^ = 0.43, *p* < 0.001), children's RSA-stressor acted as an indirect effect in explaining the link between child RF and RSA-recovery (point estimate = 0.21, 95% CI [0.03, 0.42]). Controlling for the indirect effect, the direct effect between child RF and RSA-recovery was not significant (point estimate = 0.16, 95% CI [−0.004, 0.33]).

### Hypothesis 3. association between children's RF and RSA moderated by attachment

**Figure 2:**
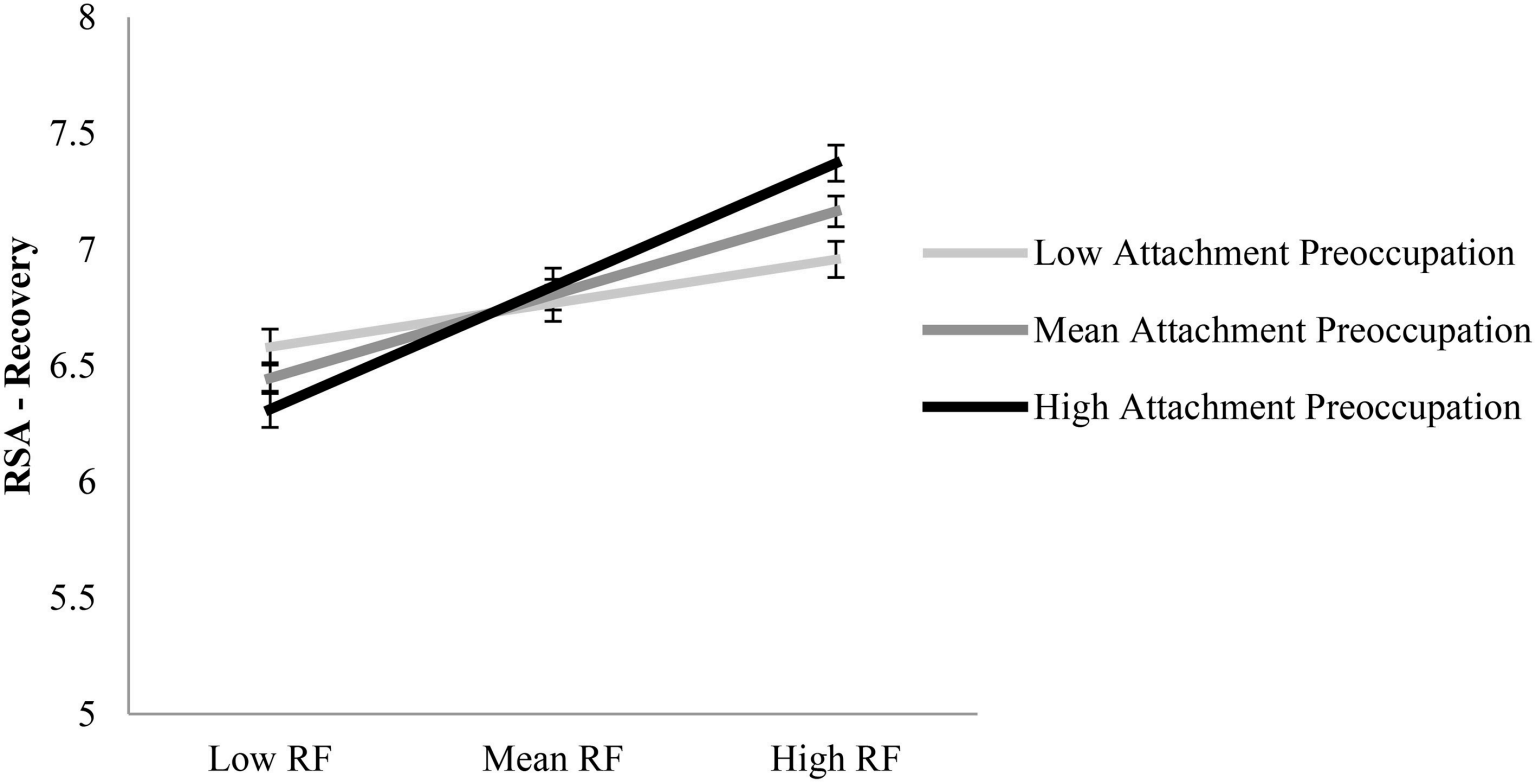
Attachment preoccupation moderates the association between children's RF and RSA-recovery, but not RSA-reactivity. RF, Reflective functioning; Attachment preoccupation, Factor analytically derived preoccupied attachment.

We tested whether attachment dismissal or preoccupation moderated the link between children's RF and RSA-stressor (Hypothesis 2a) or RSA-recovery (Hypothesis 2b), after controlling for covariates. The results of these moderation analyses revealed that neither attachment dismissal (Δ*R*^2^ = 0.01, *b* = −0.13 *p* = 0.21), nor attachment preoccupation (Δ*R*^2^ = 0.01, *b* = 0.09, *p* = 0.28), moderated the link between children's RF and RSA-stressor. However, after controlling for children's age, gender, baseline RSA, and the main effects of attachment dismissal and preoccupation (*R*^2^ = 0.48, *p* < 0.001), attachment preoccupation moderated the link between children's RF and RSA-recovery (Δ*R*^2^ = 0.05, *b* = 0.21, *p* = 0.01). Among children with mean (*b* = 0.43, *p* = 0.002), and high levels of attachment preoccupation (*b* = 0.63, *p* = 0.0001), RF was positively associated with RSA-recovery. Among children with low attachment preoccupation, the association between children's RF and RSA-recovery was not significant (*b* = 0.23, *p* = 0.15; see Figure 2). Attachment dismissal did not moderate the association between child RF and RSA recovery after the stressor task (Δ*R*^2^ = 0.004, *b* = −0.07, *p* = 0.52); therefore, we elected not to examine a moderated mediation using attachment dismissal.

#### Exploratory moderated mediation

**Figure 3:**
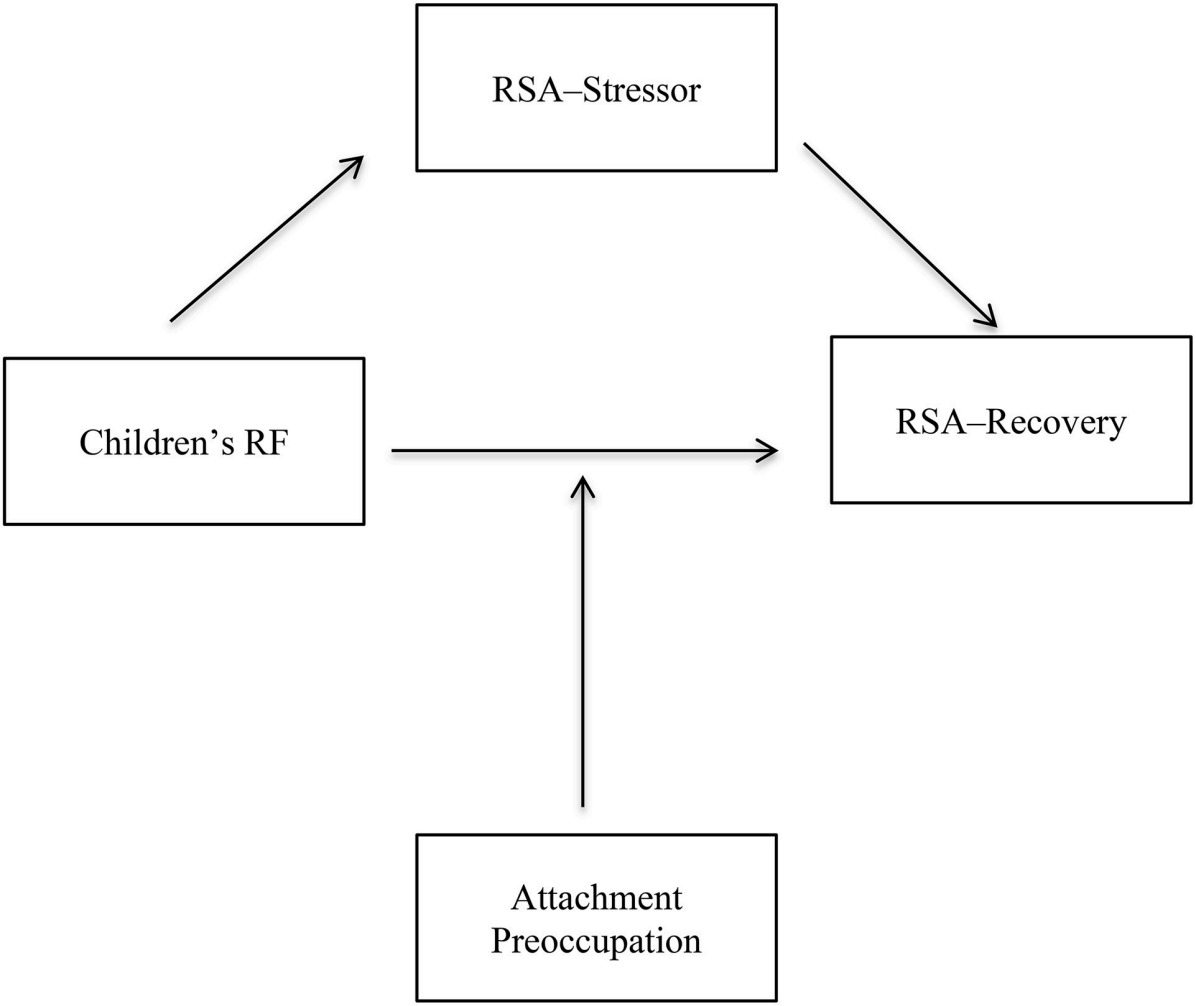
Visual depiction of the proposed moderated mediation: Attachment preoccupation moderates the association between children's RF and RSA-recovery mediated by RSA-stressor. RF, Reflective functioning; Attachment preoccupation, Factor analytically derived preoccupied attachment score (Child Attachment Interview); Higher score means highly preoccupied.

After controlling for children's age, gender, baseline RSA, and attachment dismissal (*R*^2^ = 0.43, *p* < 0.001), the examination of conditional effects revealed that among children with mean (*b* = 0.22, *p* = 0.02) and high attachment preoccupation (*b* = 0.36, *p* = 0.002) RSA-stressor mediated the link between children's RF and RSA-recovery (point estimate = 0.20, 95% CI [0.003, 0.39]; see Table 5 and Figure 3). Among children with low attachment preoccupation, there was no significant mediation effect (*b* = 0.08 *p* = 0.45).

**Table 5:** Regressions examining the moderated mediation model: attachment preoccupation as a moderator of the mediation of the children's RF to RSA-recovery by RSA-stressor.

	**Independent variable: children's RF**		
	**Dependent variable: RSA–recovery**		
**Predictor variables**	***b***	***SE***	**CI**
Low attachment preoccupation	0.08	0.11	[−0.13, 0.30]
Mean attachment preoccupation	0.22^*^	0.09	[0.03, 0.41]
High attachment preoccupation	0.36^**^	0.11	[0.14, 0.59]

*RF, Reflective functioning; Attachment preoccupation, Factor analytically derived preoccupied attachment score (Child Attachment Interview); Higher score means highly preoccupied*.

* *p < 0.05*,

** *p < 0.01*.

## Discussion

Despite extensive theorizing regarding the regulatory role of RF, prior to the current investigation, extremely few studies had explored the links between RF and physiological reactivity or regulation, and none had examined these associations in children. In the current study, we tested these associations by exploring children's physiological responses to a standardized paradigm designed to evoke reactions regarding the experience and expression of attachment-related needs. Consistent with theory and hypotheses, the key findings of this study were that higher RF was associated with lower cardiovascular reactivity and better regulation, links that were especially strong for children with greater attachment preoccupation.

The findings of our first analysis revealed that greater attachment dismissal was associated with lower RF, but that attachment preoccupation was not significantly associated with child RF. Thus, using ratings derived from independent teams of coders, we found that children classified as having high attachment dismissal had lower RF, demonstrating the limitations in their abilities to hold in mind their own and their parents' mental states. However, our findings revealing a unique link between dismissing attachment and RF are in line with results from other studies finding a specific association between dismissing attachment and lower RF in children and parents [Bizzi et al., under revision; (73), but see (36), for an association between lower RF with dismissal and preoccupation]. One potential reason for the lack of association between RF and preoccupation is that preoccupied children may use more emotion words than dismissing children, and may potentially use comparable numbers of emotion words as secure children. The use of emotion words constitutes part of the global RF score and thus could inflate the RF ratings of preoccupied children, despite their relatively infrequent use of attributions or actual mentalization. However, for preoccupied children, the use of mental state language may not be as emotionally regulating as it is for secure children, or perhaps preoccupied children use mental state language but do not achieve higher levels of mentalization (e.g., drawing connections between mental states and behavior). An alternate explanation is that preoccupied children, who are likely to be more open to experiencing and expressing negative emotion than dismissing children (16), may engage in a type of hypermentalizing in an attempt to regulate emotion, but are unable to use their mentalization in an organized way to regulate and contain negative effects and anger in relation to attachment figures (Bizzi et al., under revision).

Our central study hypotheses concerned the interrelations of RF and physiological reactivity and regulation, operationalized as RSA during the task and the recovery period, respectively. RF was associated with higher RSA during the stressor and recovery period, supporting our hypotheses and suggesting that RF is associated with lower reactivity and better regulation. Interpreting these findings in terms of theory would suggest that for children with higher RF on the CAI, contemplating attachment needs (being physically hurt, sick, sad, or frightened) did not require as much physiological regulatory effort as this task demanded from children with lower levels of RF. Thus, at least in the context of the current attachment-based task, RF was associated with superior physiological regulation. According to mentalization theory, a child's experience of the benign interest of parents in their subjective experience can improve emotion regulation, as it opens a space where they can communicate their concerns, fears, and difficulties to their parents, allowing them to develop a mutually elaborated understanding of themselves and their emotions (26, 33, 60).

However, much remains to be understood about these effects as, given the correlational design in which measures were assessed at a single timepoint, causal conclusions elude us. Further, RF could be associated with emotion reactivity and regulation via several channels. For instance, children may have been less reactive owing to a sense of confidence that emotions can be safely experienced and shared. Higher RF may in part be associated with lower stress activation because of early experiences in which children's subjective experience was responded to first through marked affect mirroring when they were young (24) and later through the parents' creation of a shared mental space where the children's subjective experience can be elaborated. Further, interactions in which the parent actively helped the child understand affective experience could have positively impacted the development of the child's stress regulation system. Alternatively, higher RF youth may not have found the vignettes emotionally taxing, as, by virtue of their abilities to mentalize, contemplating attachment needs and emotions may not be as daunting. Via mentalization, children have learned to mentally represent emotions, symbolize subjective experience, and put these experiences into words, a process which facilitates the understanding and regulation of emotions. When children develop symbolic and semantic representations of emotion, children's neurobiological pathways of stress regulation and mentalization may be more effectively connected, thus promoting the effective regulation of emotion. We tentatively suggest that the outcome of interpersonally developed mentalization about self and others, evident at the level of physiological regulation, may reflect an integration of symbolic and affective processes that are likely evident at the level of neurobiology but may also be seen from the perspective of self and identity. At this level of development, higher RF may be seen as an index of the child's emerging sense of self and attachment figures, and as central to identity.

Finally, we found that attachment preoccupation, but not attachment dismissal, moderated the link between RF and RSA during recovery, but not RSA reactivity. Specifically, the positive association between RF and RSA was only statistically significant among children with mean or higher attachment preoccupation, and not among children with low levels of attachment preoccupation. Children who are low in attachment preoccupation may not need RF to regulate themselves when considering attachment needs and feelings, as contemplating these topics may have evoked less intense reactions. On the other hand, for preoccupied children, for whom the contemplation of attachment needs may have caused higher reactivity, RF appears to have helped in their recovery. We have previously speculated whether the higher measured RF found among preoccupied children was indicative of hypermentalizing (repetitive, unproductive contemplating about mental states), but the current findings suggest that RF does in fact facilitate regulation among preoccupied children.

This finding can be understood in terms of its contribution to the notion that RF promotes resilience—children whose mental representations are characterized by preoccupation, who were nonetheless simultaneously engaged in the process of making sense of these experiences (evidenced by high RF), demonstrated superior physiological recovery from the stressor task. As resilience can be conceptualized as the capacity of a system to adjust to disturbances that could threaten it, or the attempt to continually derive meaning and insight from experiences (62), it aptly characterizes the process of mentalization co-occurring with preoccupied attachment. The link between RF and physiological reactivity did not vary as a function of dismissing attachment; at all levels of dismissing attachment, higher RF was associated with better emotion reactivity and regulation. Thus, no matter how low the attachment dismissal, RF confers regulatory protection.

### Strengths and limitations

As the first empirical test of the links between RF and physiological reactivity in children, we believe that this study contributes to the literature in significant ways. By using robust observational measures of attachment and RF, and by employing a standardized laboratory stressor designed to present to children situations in which attachment needs are evoked, we offer an important, highly controlled examination of research hypotheses. Further, our use of a highly racially and ethnically diverse sample of children increases the generalizability of the findings we report.

However, it is also important to contextualize the contributions of this study in light of its limitations. One limitation of the study is that the assessment occurred at a single timepoint, leaving open the possibility that lower physiological reactivity or better regulation could cause higher levels of RF, or that a shared third variable drives the association between RF and emotion reactivity and regulation. Longitudinal designs will be able to identify whether RF predicts emotion regulation later in development, which would strengthen the argument that children's RF promotes resilience. Further, we examined attachment and RF using the same instrument (CAI). Although we used non-overlapping coders who were blind to all participant information, the fact that these indices were derived from the same measure may limit the extent to which we can accurately conceptualize them as separable constructs.

Further, measuring children's RSA in response to the distress vignettes task did not permit us to examine the types of negative emotion reactivity and regulation that are associated with RF (e.g., we cannot make an argument regarding discrete emotions), nor can we speak to children's subjective emotional experience more generally; this is an area ripe for future inquiry. Relatedly, for the purposes of this study, we attempted to distinguish between physiological measures of reactivity (RSA measured during the presentation of the vignettes) and regulation (RSA measured during the 30 s following each vignette block); however, this distinction contains some error in that children can employ regulation before and during the presentation of the distress vignettes. Thus, it is impossible to conclude that these measures indexed reactivity and regulation, but we can state that they assessed early and later measures of cardiovascular activation. In terms of the use of RSA, we note that some researchers suggest that greater decreases in RSA during a demanding task connote greater activation to stimuli and thus more optimal use of coping strategies (78–80), while others suggest that higher RSA during a stressor signifies lower emotional reactivity, which serves an adaptive function (10–12). Thus, it is important to note that our interpretation that lower stressor- and recovery-RSA signifies greater reactivity is consistent with one way of conceptualizing task-related changes of RSA, but this view is not universally held.

In addition, we did not measure or control for children's reading or learning abilities, which leaves open the possibility that the effects found here are biased to some extent by children's cognitive functioning. Finally, in future studies, it would be informative to measure task-specific or state-like RF regarding the laboratory task, as has been done in at least one other investigation (46), as this would enable us to get closer to identifying the processes occurring during the stressor task for high RF children.

## Conclusions

This study provides new physiological evidence that children's mentalization is associated with more efficient stress regulation, as higher RF was associated with less physiological reactivity during and more efficient recovery from a stressor. When confronted with attachment stress, children with higher RF regulated their autonomic nervous systems with less effort than children with lower RF. Consistent with the argument that RF promotes resilience, the association between RF and higher RSA was only significant among children with higher levels of attachment preoccupation.

## Author contributions

JB designed the study, developed the hypotheses, oversaw the data collection, conducted data analyses, and was the chief contributor to the writing of the manuscript. KE developed the coding system for children's reflective functioning, oversaw the coding of the data, and contributed to the writing and editing of the manuscript. KH assisted with data analyses, editing and writing of the manuscript, and the preparation and checking of references. ATS conducted the coding of the reflective functioning data and assisted with the literature review of the manuscript. RD provided conceptual guidance regarding the framing of the manuscript. PF assisted with the conceptual framing of the mentalization aspect of the manuscript.

### Conflict of interest statement

The authors declare that the research was conducted in the absence of any commercial or financial relationships that could be construed as a potential conflict of interest. The reviewer DRO and handling Editor declared their shared affiliation.
